# Improved HPLC Method for the Determination of Moxifloxacin in Application to a Pharmacokinetics Study in Patients with Infectious Diseases

**DOI:** 10.1155/2013/462918

**Published:** 2013-05-26

**Authors:** Nan Wang, Liqin Zhu, Xuequn Zhao, Wenjie Yang, He Sun

**Affiliations:** ^1^Pharmacy Department, The Third Central Hospital, No. 83 Jintang Road, Hedong District, Tianjin 300170, China; ^2^Basic Medical Department, Tianjin Medical University, No. 22 Qixiangtai Road, Heping District, Tianjin 300070, China; ^3^Pharmacy Department, First Center Hospital, No. 24 Fukang Road, Nankai District, Tianjin 300192, China; ^4^Infectious Disease Department, First Center Hospital, Tianjin, China; ^5^School of Pharmaceutical Science and Technology, Tianjin University, No. 92 Weijin Road, Nankai District, Tianjin 300072, China

## Abstract

*Objective*. To develop a simple and rapid high-performance liquid chromatography (HPLC) method for measuring moxifloxacin concentration in human plasma. *Methods*. Following a single step liquid-liquid extraction, analytes along with an internal standard (IS) were separated using an isocratic mobile phase of 0.1% triethylamine (adjusted pH to 4.8 with phosphoric acid)/acetonitrile (80/20, v/v) at flow rate of 1 mL/min on reverse phase Kromasil C_18_ column (250 mm × 4.6 mm, 5 **μ**m) at room temperature. *Results*. Total analytical run time for selecting moxifloxacin was 15 min. The assays exhibited good linearity (*r* ^2^ = 0.9998) over the studied range of 25 to 5000 ng/mL. The absolute recovery rate of low, medium, and high concentrations were 69.88%, 78.86%, and 78.51%, respectively. The relative recovery rates were 98.50%, 96.61%, and 101.79%, respectively. Coefficient of variation and error at both of the intraday and interday assessments were less than 4.7%. *Conclusions*. The results indicated that this method is a simple, rapid, precise and accurate assay for the determination of moxifloxacin concentrations in human plasma. This validated method is sensitive and reproducible enough to be used in pharmacokinetic studies.

## 1. Introduction

Moxifloxacin is a fourth-generation fluoroquinolone antibiotic that exerts its effects by trapping a DNA drug enzyme complex and specifically inhibiting ATP-dependent enzymes topoisomerase II (DNA gyrase) and topoisomerase IV. Currently, moxifloxacin is being extensively used in the treatment of respiratory system diseases such as community-acquired pneumonia (CAP), chronic bronchitis (CB) and chronic obstructive pulmonary disease (COPD) for the broad spectrum of antimicrobial activity against respiratory tract pathogens, including Gram-positive and Gram negative organisms, anaerobic bacteria, and atypical respiratory tract pathogens [1–4]. The favorable pharmacokinetics of moxifloxacin, including a high mean apparent volume of distribution and a long terminal half life, supports a once-daily dosing regimen in the treatment of infectious disease [5]. It is revealed that moxifloxacin is primarily eliminated in the liver [6].

In recent years, a variety of methods on high-performance liquid chromatography (HPLC) for measuring moxifloxacin concentration in plasma have been reported. Fluorescence detector was applied in several methods for its advantage of sensitivity [7–12]. However, some complex techniques such as gradient elution and on-column focusing [7], precolumn derivatisation [8], or special column [9], were employed. In addition, these expensive specific instruments would increase the experiment cost and not brief enough for clinical application. Although a few methods applied HPLC with UV detector to determine moxifloxacin in plasma [13–16], automated extraction methods with a polymeric cartridge [13], poor extraction recovery [14], or complicated flow phase [15] were involved. LC/ESI-MS/MS methods have also been reported [17, 18], but these advanced techniques are not suitable for clinical routine.

In this paper, we describe a rapid and simple HPLC method for determination of moxifloxacin in human plasma using ultraviolet (UV) detection that allows rapid processing of large number of plasma samples. The method employed a simple mobile phase of weak acidity, permitting the stability of moxifloxacin, and the application of Kromasil C_18_ column ensures the efficient separation of the drug. Ciprofloxacin was used as IS in the method, which exhibit goods separation of the two kinds of fluoroquinolones.

## 2. Materials and Methods

### 2.1. Chemicals

Pure moxifloxacin powder and ciprofloxacin lactate injection were purchased from Bayer, China. HPLC grade acetonitrile, methanol (Tianjin Xiehe Haopeng Chromatography Technology Co., Ltd., China), triethylamine (Tianjin Bodi Chemical Industry Co., Ltd., China), and phosphoric acid (Shijiazhuang Chemical Factory, China) were used for the HPLC analysis. Pooled human plasma was obtained from Tianjin Blood Center, China.

### 2.2. Apparatus and Chromatographic Conditions

The HPLC system (Shimadzu Chromatography Division, Japan) consisted of a pump (LC-10AT), a UV detector (Shimadzu, SPD-10AVP Plus), and autosampler with built-in system controller. Analysis Star workstation was used for data collection and acquisition. Chromatographic separation of moxifloxacin was achieved using a Kromasil C_18_ (250 mm × 4.6 mm, 5 *μ*m) reverse phase analytical column.

The mobile phase consists of a mixture of 0.1% triethylamine (adjusted pH to 4.8 with phosphoric acid)/acetonitrile (80/20, v/v). The mobile phase was pumped at an isocratic flow rate of 1 mL/min at room temperature. The wavelength of UV detection was set at 296 nm for moxifloxacin.

### 2.3. Preparation of Standard Solutions

A stock solution (5 *μ*g/mL) of moxifloxacin was prepared by dissolving 500 *μ*g of drugs in 100 mL of mobile phase. Working solutions were prepared from the stock solution by sequential dilution with mobile phase just before use. All solutions were protected from light by covering them with aluminium foil and stored in brown volumetric flask at 4°C. Ciprofloxacin was used as IS. The stock solution (5 *μ*g/mL) of ciprofloxacin was prepared.

### 2.4. Extraction Procedure

Plasma samples 200 *μ*L were transferred to a 1.5 mL centrifuge tube, and then 40 *μ*L of IS stock solution (5 *μ*g/mL) was spiked. The sample was vortexed for 30 second. Then, 1 mL acetonitrile was added. After being vortexed for 3 min, the sample was centrifuged for 10 min at 13000 rpm. The organic layer was transferred to another centrifuge tube and evaporated at 40°C under a stream of air. Then, the dried extract was reconstituted with 200 *μ*L of mobile phase, and a 60 *μ*L aliquot was injected into chromatographic system. 

### 2.5. Assay Selectivity

Selectivity of the assay method was assessed by evaluating potential interference from endogenous compounds in the blank plasma as well as the separation of moxifloxacin and IS. The separation efficiency of moxifloxacin and IS and probable endogenous compounds from plasma was checked by comparing the chromatograms of blank plasma, blank plasma with IS, and blank plasma with IS plus moxifloxacin. The samples were extracted and analyzed as described above. The peak and the retention time for each drug under the chromatographic conditions of the moxifloxacin assay were recorded. In addition, chromatogram of pure standard analyte and IS in mobile phase was evaluated as a control.

### 2.6. Calibration Curve

To 200 *μ*L of blank human plasma 1, 2, 4, 20, 40, 100, and 200 *μ*L of moxifloxacin working solution were added, yielding final concentrations of 0.025, 0.05, 0.1, 0.5, 1.0, 2.5, and 5.0 *μ*g/mL moxifloxacin. To this mixture, 40 *μ*L of IS working solution was added to yield IS concentration of 1.0 *μ*g/mL. Calibration samples were prepared for analysis as described above. Each concentration point in the calibration curve was analyzed three times under the same HPLC conditions as described above. The peak area ratios of moxifloxacin to the IS for each of the standard solutions were calculated and plotted as a function of drug concentrations in human plasma. The calibration curves were acceptable only if they had correlation coefficients (*r* ^2^) of 0.99 or greater.

### 2.7. Repeatability and Precision

Repeatability was evaluated at 0.5 *μ*g/mL of moxifloxacin with 1.0 *μ*g/mL of IS in 10 mL flow phase. This was measured for six times per day and the relative standard deviation (RSD) was calculated.

To examine the precision of the method, plasma spiked with three concentrations consisting of low, middle, and high concentrations (0.05, 0.5, and 2.5 *μ*g/mL) of the analyte was prepared. Intra-day precision was evaluated by analyzing the spiked controls five times a day. This was repeated on five consecutive days to permit an assessment of inter-day precision.

### 2.8. Extraction Recovery

Recovery of moxifloxacin was determined by comparing the peak area of the analyte extracted from the plasma with peak area obtained by the direct injection of pure standard analyte in mobile phase at three different concentrations containing low, middle and high concentrations (0.05, 0.5, and 2.5 *μ*g/mL).

### 2.9. Method Recovery

Moxifloxacin stoking solution 2, 20, and 100 *μ*L was added to 200 *μ*L blank plasma, respectively; then 40 mL IS working solution was added. Following the Extraction procedure, the sample was prepared and then determined for five times for each concentration. The concentration of moxifloxacin was obtained by putting the peak area ratio into the calibration curve. The method recovery was calculated by comparing the determined concentration and the actual concentration.

### 2.10. Application to Pharmacokinetic Study

The validated HPLC method was applied in a population pharmacokinetics research of moxifloxacin in 37 Chinese adult patients with infectious disease including CAP, CB, urinary tract infection (UTI), and bacterial diarrhea. The patients were recruited after obtaining informed consent. Moxifloxacin was administered as a single intravenous dose of 400 mg once daily for 5–14 days. Two venous blood samples (2 mL) were collected into heparinised tube right after the first administration and before the fourth dose. The blood samples were immediately centrifuged (3500 rpm, 10 min) to separate and stored at −80°C until assayed. The index of age, gender, height, weight, blood creatinine (Cr), urea nitrogen (BUN), glutamic-pyruvic transaminase (ALT), and glutamic-oxalacetic transaminase (AST) concentration was recorded. A correlation between moxifloxacin blood concentration and patients' individual data was studied. 

## 3. Results

### 3.1. Assay Selectivity

**Figure 1:**
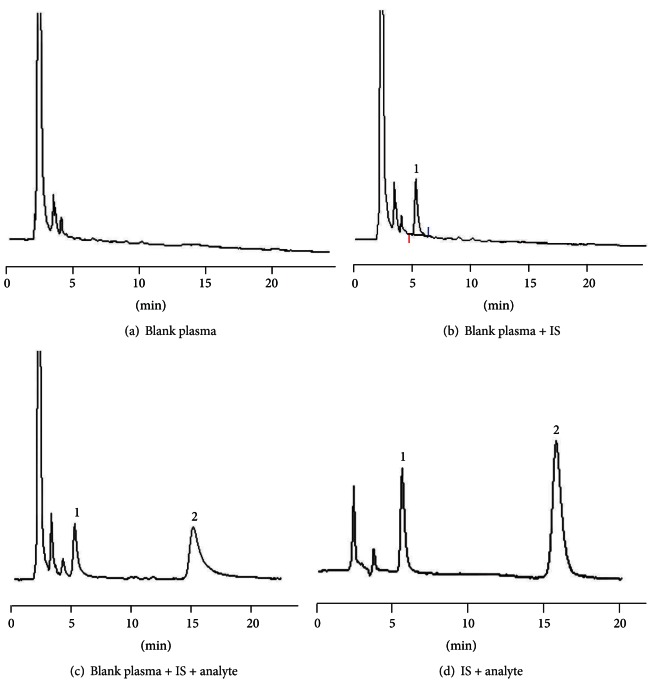
Chromatogram. 1: IS (ciprofloxacin). 2: moxifloxacin.

The selectivity of the assay was tested.  Figure 1(a) was a representative chromatogram of a blank plasma sample. The chromatogram showed no interfering peaks near the same retention times as moxifloxacin or internal standard derivatives (Figures 1(b) and 1(c)). The chromatographic peaks were well resolved to baseline.  Figure 1(d) was the chromatogram of pure standard analyte and IS in mobile phase, which demonstrated the peaks in Figures 1(b) and 1(c), was derived from moxifloxacin and IS.

### 3.2. Linearity of Calibration Curve

**Figure 2:**
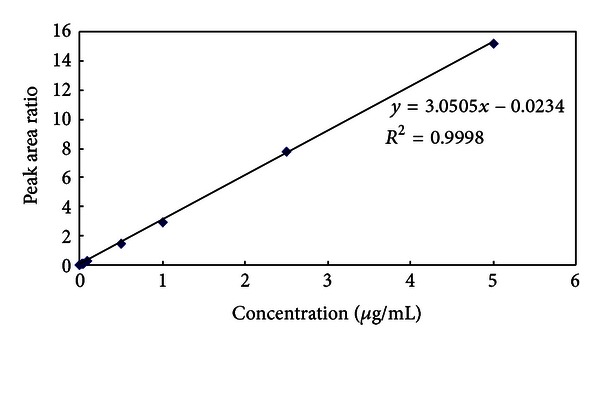
Calibration curve of moxifloxacin in plasma (*n* = 5).

The linearity of the calibration curve for moxifloxacin in spiked drug-free plasma over the concentration range of 25–5000 ng/mL was evaluated. The peak area ratio of each concentration in determining the calibration curve was exhibited in Table 1. Calibration curve obtained by plotting peak area ratio (moxifloxacin/internal standard) versus concentration was linear over the range of 25–5000 ng/mL. The calibration curve of moxifloxacin in plasma was linear and was represented by the regression equations *y* = 3.0505*x* − 0.0234 (Figure 2), where *y* presents the peak area ratio (moxifloxacin/IS) and *x* represents the plasma concentration of moxifloxacin (*μ*g/mL). The mean correlation coefficient (*r* ^2^) for the moxifloxacin calibration curves was 0.9998.

### 3.3. Repeatability and Precision

The results of repeatability tests were displayed in Table 2. RSD of repeatability was 1.31%.

Intraassay, interassay precision data were summarized in Tables 3 and 4. The intra-assay RSDs at the three concentrations were 4.12, 3.12, and 1.85, while the inter-assay RSDs were 4.70, 1.16, and 3.38. These results indicated that the validated assay was precise, accurate, and reproducible.

### 3.4. Extraction Recovery

The mean percentage extraction recoveries of moxifloxacin at the three concentrations were 69.88 ± 3.19, 78.86 ± 4.12, and 78.51 ± 2.44, respectively (Table 5). The use of IS, namely, ciprofloxacin, was crucial to decrease the error brought by the extraction procedure. In this study, ciprofloxacin was used as IS because the structure and properties of ciprofloxacin were similar to moxifloxacin. Ciprofloxacin exhibits good ultraviolet response at 278 nm wavelength.

### 3.5. Method Recovery

Mean value of the method recovery was 98.85%, which ranged from 96.6 to 101.8 (Table 6).

Application of the method to a population pharmacokinetics study in patients with infectious diseases.

To demonstrate its utility in a pharmacokinetics study, the method was applied to measure the concentrations of moxifloxacin in plasma samples obtained from patients with infectious disease who were given intravenous moxifloxacin 400 mg once daily. Peak moxifloxacin concentrations in plasma ranging from 1.652 to 6.743 *μ*g/mL with an average of 3.364 ± 1.686 *μ*g/mL were achieved right after intravenous administration of moxifloxacin. Trough concentrations ranged from 0.359 to 2.676 *μ*g/mL with an average of 0.914 ± 0.481 *μ*g/mL. 

## 4. Discussion

### 4.1. Optimization of HPLC Chromatographic Conditions

The present study to determine the blood concentration in plasma principally focused on HPLC with UV detection, on account of its low sample amounts, easy operation, and high sensitivity. However, tetrabutylammonium hydrogen sulfate, which was expensive and not easy to get, has to be used as ion-pair reagent. In addition, fluorescence detector itself was expensive. Moxifloxacin, which had strong ultraviolet absorption, could be absorbed in 296 nm. Therefore, HPLC-UV method was applied in this experiment, which was more economical. Internal standard method was adopted in order to reduce the system error caused by the instruments and operations. Ciprofloxacin was used as internal standard. This method was easy to operate, simple in sample preparation, and highly sensitive. 

### 4.2. Flow Phase

In the pretest, the solutions of acetonitrile—0.01 mol/L potassium dihydrogen phosphate and 0.01 mol/L potassium dihydrogen phosphate-methanol-acetonitrile—were used as flow phase. However, peaks more than expected were observed leading to uncertain drug peak due to unknown reason. Later, 1% triethylamine-acetonitrile was attempted and got ideal separation. Acetonitrile was the liquid that influenced retention behavior of moxifloxacin. The retention time of moxifloxacin would be shortened as the proportion of acetonitrile increased.

Retention time also varied with pH value. The higher pH value was, the longer the retention time of moxifloxacin would be. Moxifloxacin did not separate completely with endogenous substances in plasma when pH value of flow phase was 4.5. The problem was solved after pH value was increased to 4.8.

According to the results above, 0.1% triethylamine (adjusted pH to 4.8 with phosphoric acid)/acetonitrile (80/20, v/v) were confirmed as flow phase which exhibited good separation among compounds in plasma, IS, and moxifloxacin with a relatively short retention time. 

### 4.3. Internal Standard

Internal standard method was adopted in order to reduce the system error caused by the instruments. In this study, we were aiming to find an internal standard that exhibited good separation with moxifloxacin and could be obtained as easy as possible. Some previous methods used internal standards which were difficult to obtain commercially [8, 15]. In this study, ciprofloxacin was used as IS and the retention time was about 5 min, which allowed moxifloxacin to separate completely. Therefore, a more suitable method having a wider range of capability is required for evaluating the precise pharmacokinetics of these drugs.

### 4.4. Determination Result Analysis

Moxifloxacin concentrations of 37 patients with a continuous intravenous dose of 400 mg once daily were determined in the tests. Peak concentration in the first dose and trough concentration in the fourth day were studied, respectively. According to the results, liver function would be responsible for the peak concentration while age and renal function may influence the trough concentration of the medicine, although the number of patients in the study was limited.

Barth et al. [19] found the pharmacokinetic feature varied little between normal and liver dysfunction patients, so that it was not necessary to adjust treatment dosage in patients with liver damage. In this study, the peak concentration of No1 patient (weight as 45 kg, ALT, AST as 53.8 and 39.8, resp.), 4.116 *μ*g/mL, may lead to the conclusion that liver dysfunction may increase the peak concentration.

Moxifloxacin would be metabolized to M1 and M2 after the second phase biotransformation and be proved not to increase as the age of the patients is growing [1, 6]. 

Skalioti et al. [20] demonstrated the plasma concentrations of moxifloxacin in patients undergoing continuous ambulatory peritoneal dialysis were 5.86 ± 0.60 mg/L, 1 hour after an oral dose of 400 mg, which was higher than the concentrations in healthy patients, 2.5–3.4 mg/L, leading to a conclusion that renal dysfunction may influence the metabolism of moxifloxacin and increase the concentration.

## 5. Conclusion

In this study, a new HPLC-UV method has been developed. The method validation indicates that this method is a simple, rapid, precise, and accurate assay for the determination of moxifloxacin concentrations in human plasma. Moreover, the method requires only a small volume (200 *μ*L) of plasma, which makes it suitable for studying the pharmacokinetics in patients, especially for the elderly. The sensitivity and simplicity of the method makes it suitable for routine therapeutic drug monitoring or clinical pharmacokinetic studies of moxifloxacin. In conclusion, this optimized method is sensitive and reproducible enough to be used in pharmacokinetic studies. 

## Figures and Tables

**Table 1:** Calibration curve (*n* = 5, $\overset{-}{x}\pm s$).

Actual concentration (*μ*g/mL)	Peak area ratio
0	0
0.025	0.098 ± 0.007
0.05	0.129 ± 0.006
0.1	0.304 ± 0.016
0.5	1.452 ± 0.030
1.0	2.901 ± 0.042
2.5	7.740 ± 0.249
5.0	15.190 ± 0.194

**Table 2:** Repeatability (*n* = 6).

Actual concentration (*μ*g/mL)	Peak area ratio	$\overset{-}{x}\pm s$	RSD (%)
0.5	1.507	1.496 ± 0.020	1.31
	1.477		
	1.485		
	1.496		
	1.483		
	1.530		

**Table 3:** Intraday precision *n* = 5, $\overset{-}{x}\pm s$).

Actual concentration (*μ*g/mL)	Determined concentration (*μ*g/mL)	Peak area ratio	RSD (%)
0.05	0.049 ± 0.002	0.129 ± 0.005	4.12
0.5	0.480 ± 0.022	1.442 ± 0.045	3.12
2.5	2.632 ± 0.056	8.007 ± 0.148	1.85

**Table 4:** Interday precision (*n* = 5, $\overset{-}{x}\pm s$).

Actual concentration (*μ*g/mL)	Determined concentration (*μ*g/mL)	Peak area ratio	RSD (%)
0.05	0.050 ± 0.003	0.131 ± 0.006	4.70
0.5	0.481 ± 0.008	1.447 ± 0.017	1.16
2.5	2.537 ± 0.127	7.717 ± 0.261	3.38

**Table 5:** Extraction recovery (*n* = 5).

Actual concentration (*μ*g/mL)	Peak area obtained by the direct injection of pure standard analyte in mobile phase	Peak area of the analyte extracted from the plasma	Extraction recovery (%)
0.05	16211	11328 ± 517	69.88 ± 3.19
0.5	161033	126989 ± 6632	78.86 ± 4.12
2.5	857954	673543 ± 20935	78.51 ± 2.44

**Table 6:** Method recovery (*n* = 5, $\overset{-}{x}\pm s$).

Actual concentration (*μ*g/mL)	Determined concentration (*μ*g/mL)	Method recovery (%)	RSD (%)
0.05	0.049 ± 0.002	98.50 ± 3.95	4.01
0.5	0.483 ± 0.010	96.61 ± 1.98	2.05
2.5	2.545 ± 0.082	101.79 ± 3.26	3.21
